# Clinical features and management of post-necrotizing enterocolitis strictures in infants: A multicentre retrospective study

**DOI:** 10.1097/MD.0000000000020209

**Published:** 2020-05-08

**Authors:** Wei Liu, Yi Wang, Jin Zhu, Chi Zhang, Guobin Liu, Xin Wang, Yanhui Sun, Zhenhua Guo

**Affiliations:** a Department of Neonatal Surgery; Ministry of Education Key Laboratory of Child Development and Disorders; National Clinical Research Center for Child Health and Disorders (Chongqing); China International Science and Technology Cooperation base of Child development and Critical Disorders; Chongqing Key Laboratory of Pediatrics; Children's Hospital of Chongqing Medical University, Chongqing; b Department of general Surgery, Children's Hospital of Shenzhen, Shenzhen; c Department of Neonatal Surgery, Maternal and child health hospital in Zunyi, Zunyi; d Department of Neonatal Surgery, Maternal and child health hospital in Chongqing, Chongqing, PR China.

**Keywords:** necrotizing enterocolitis, neonatal, post-NEC strictures

## Abstract

To explore the clinical features and management of post-necrotizing enterocolitis strictures.

Clinical data from 158 patients with post-necrotizing enterocolitis strictures were summarized retrospectively in 4 academic pediatric surgical centers between April 2014 and January 2019. All patients were treated conservatively in the internal medicine department. All patients underwent preoperative X-ray examinations, 146 patients underwent gastrointestinal contrast studies, and 138 patients underwent rectal mucosal biopsies. All of the patients were treated surgically.

Of the 158 patients, 40 of them had necrotizing enterocolitis (NEC) Bell stage Ib, 104 had Bell stage IIa, and 14 had Bell stage IIb. In these patients, the clinical signs of intestinal strictures occurred at mean of 47.8 days after NEC. In 158 patients, 146 underwent barium enema examination, 116 demonstrated intestinal strictures, and 10 demonstrated microcolon and poor development. A total of 138 patients underwent rectal mucosal biopsies, and 5 patients had Hirschsprung disease. Intraoperative exploration showed that intestinal post-NEC strictures occurred in the ileal (17.7%, 28/158) and colon (82.3%, 130/158), including ascending colon, transverse colon and descending colon, and multiple strictures were detected in 36.1% (57/158) patients. Surgical resection of stricture segments in the intestine and primary end-to-end anastomosis were performed in 142 patients, and the remaining 16 patients underwent staged surgeries. In the 146 patients with complete follow-up data, 9 had postoperative adhesions: 4 of them received conservative treatment, and the others underwent a second operation. Fifteen patients were hospitalized 1 to 3 times for malnutrition and dehydration due to repeated diarrhea; these patients eventually recovered and were discharged smoothly. All the other patients had uneventful recoveries without stricture recurrence.

Post-NEC strictures mostly occurred in the colon, and there were some cases of multiple strictures. A gastrointestinal contrast study was the preferred method of examination. Preoperative rectal mucosal biopsy resulted in a diagnosis of Hirschsprung disease, and then a reasonable treatment protocol was chosen. Surgical resection of stricture segments in the intestine and primary end-to-end anastomosis achieved good therapeutic effects with favorable prognoses in these patients.

## 1 Introduction

Neonatal necrotizing enterocolitis (NEC) is one of the most common acute abdominal diseases in newborns. NEC has the characteristics of an acute onset, rapid development, high mortality and many complications. The incidence of NEC in premature infants is 3.0% to 5.0%, which is mainly related to the gestational age of newborns at birth,^[1]^ and the mortality rate can be as high as 50.0%.^[2]^ With increasing attention to the disease in medical institutions and the substantial improvements in medical expertise, most childrens conditions become gradually stable after active treatment, but the frequency of late complications has increased. The late complications of NEC include intestinal strictures, cholestatic liver disease, and short bowel syndrome. Post-NEC strictures are the most common type of complication in the late stage of NEC, with an incidence ranging from 11.0% to 35.0%. The intestinal strictures complicated by NEC are secondary intestinal strictures, which are different from congenital intestinal strictures or intestinal atresia in newborns. To improve the level of clinical diagnosis and treatment, the clinical characteristics, diagnosis and management of post-NEC strictures were summarized and analyzed.

## 2 Materials and methods

### 2.1 Ethical approval and informed consent

This multi-centre study was conducted at the Childrens Hospital of Chongqing Medical University, the Maternal and Child Health Hospital in Chongqing, the Maternal and Child Health Hospital in Zunyi, and the Childrens Hospital in Shenzhen. A review of the data collection was approved by the local ethical committee in all 4 hospitals.

All guardians of the participants signed an informed consent form prior to the study and were debriefed after the assessment. All guardians of the participants were informed that participation was voluntary and that they had the right to refuse or stop participating in the study at any time.

### 2.2 Patients

A total of 158 patients with intestinal strictures were diagnosed in the 4 academic pediatric surgical centers between April 2014 and January 2019, and there were 96 males and 62 females. A total of 54 of the patients had been full-term births, and 104 were premature infants (65.8%); there was an average age of 54.6 days (12–202 d), and there were 72 normal birth weight infants and 78 low birth weight infants (<2500 g). Among these 78 infants, 8 were very low birth weight infants (<1500 g). NEC Bell staging resulted in 40 patients with stage Ib, 104 patients with stage IIa and 14 patients with stage IIb. All patients were treated conservatively in the internal medicine department. Inclusion criteria:

1. a clear history of NEC and conservative treatment with hospitalization or even many instances of conservative treatment in hospitalized children; and
2. intraoperative exploration and pathological examination with a diagnosis of intestinal strictures.

Exclusion criteria:

1. patients with gastrointestinal perforation or critical conditions who underwent emergency fistulostomy, 96 patients were excluded; and
2. the guardian of the patients did not sign the informed consent form, 57 patients were excluded.

### 2.3 Clinical manifestation

All of the 158 patients had a clear history and were diagnosed with NEC. After successful conservative treatment, the children developed varying degrees of abdominal distension (63.3%, 100/158), bloody stool (48.3%, 76/158), feeding intolerance (35.4%, 56/158), and vomiting (25.3%, 40/158).

### 2.4 Investigations

X-ray abdominal vertical lateral radiography was performed in all patients, barium enema examination was performed in 146 patients, and rectal mucosa biopsy was performed in 138 patients. The tissues were examined by immunohistochemistry, the S-P method and DAB staining. All the children received surgical treatment. One hundred forty six patients were followed up completely. A combination of outpatient follow-up and telephone follow-up was used. The main follow-up measurements were of appetite, stool, abdominal distension, diarrhea, and body mass.

## 3 Results

### 3.1 Supplementary examination

**Figure 1:**
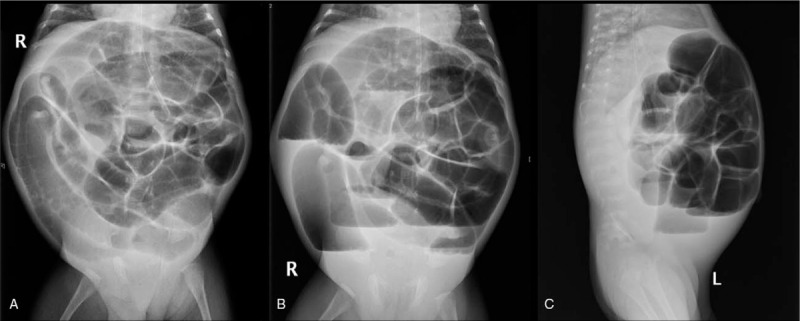
Abdominal X-ray appearance of a post-NEC stricture. A: Intestinal expansion in a decubitus film. B: Intestinal expansion and liquid level in a stand-up film. C: Fluid and dilatation of the lower ileus in a lateral film.

**Figure 2:**
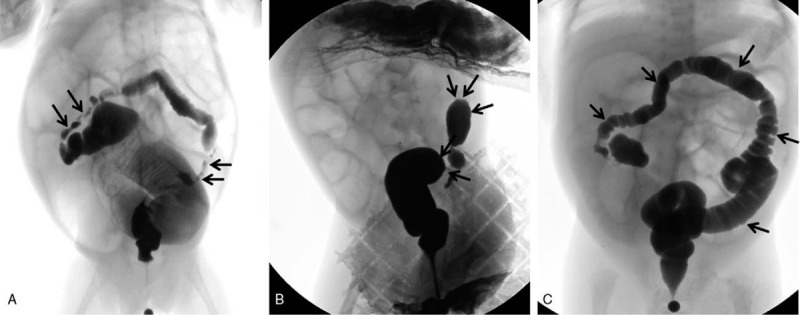
Different types of intestinal strictures with barium enema examination. A: Stricture appearance, small lumens (black arrow) and dilatation of the proximal bowel in the stricture segment. B: Blind-end appearance (black arrow), contrast agents have difficulty passing through the stricture segments, dilated proximal intestinal canal. C: Fetal colon (black arrow), all colons were small and poorly developed.

**Figure 3:**
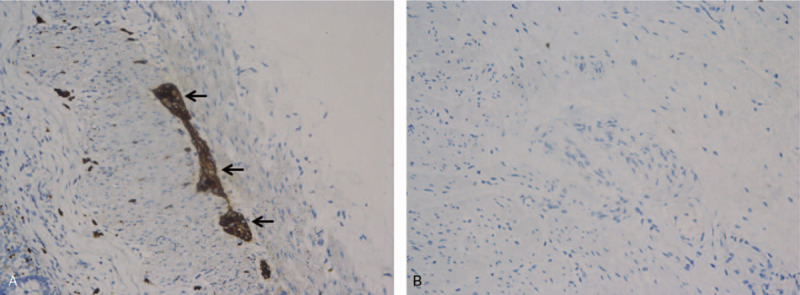
CR immunohistochemistry of rectal suction biopsy. A: Normal expression of CR-immunoreactive ganglion cells (arrow) and nerve fibers in the myenteric plexus (×200) B. No positive CR-immunoreactive ganglion cells (arrow) in the myenteric plexus of HSCR tissues (×200).

**Figure 4:**
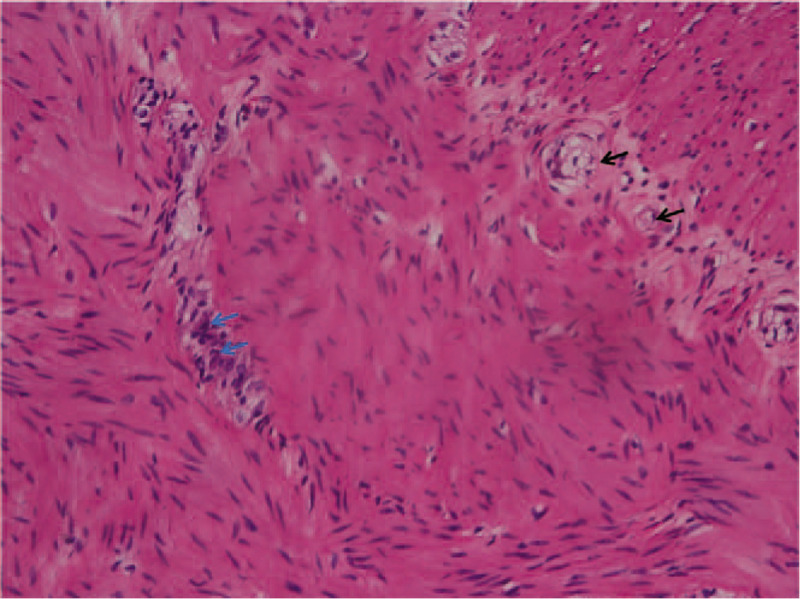
H & E staining of the stricture segment (×200). Chronic inflammatory cell infiltration (blue arrow) and poorly developed nerve cells (black arrow) in the submucosa of the intestinal wall.

X-ray examination was performed in all 158 children with post-NEC strictures, and positive signs were found in 142 children. The abdominal vertical and lateral films mainly showed intestinal dynamic changes or multiple groups of continuous inflatable intestinal curvatures, and several high and low liquid and gas levels were seen in the vertical position. Part of the intestinal space was broadened (Fig. 1). Barium enema examination was performed in 146 patients, and intestinal strictures were found in 116 patients, shown as strictures (Fig. 2A) or blind ends (Fig. 2B). No obvious abnormalities were found in 18 patients, and fetal colon was suggested in 12 patients (Fig. 2C). Rectal mucosa biopsies were performed in 138 patients, and 5 patients had no ganglion cells (Calretinin (CR) negative), which was suggestive of Hirschsprung disease (HSCR)^[3,4]^ (Fig. 3). The pathological manifestations of post-NEC strictures mainly included local tissue hyperaemia, bleeding or necrosis with acute or chronic inflammatory cell infiltration, and poorly developed nerve cells could be seen in the submucosa of the intestinal wall (Fig. 4).

### 3.2 The site of intestinal strictures

In the 158 patients, there were 28 cases of ileal strictures and 130 cases of colonic strictures, including those of the ascending colon (n = 55), hepatic flexure of the transverse colon (n = 10), transverse colon (n = 50), splenic flexure of the transverse colon (n = 17), descending colon (n = 46), and sigmoid colon (n = 29). Multiple intestinal strictures, including those with 2 or more stricture sites, were found in 57 patients.

### 3.3 Time of intestinal strictures

The time of the intestinal strictures was determined in 127 children: there were 2 patients with a time of <14 days, 15 patients with 15 to 28 d, 52 patients with 29 to 42 d, 29 patients with 43 to 56 d, 16 patients with 57 to 70 d, and 13 patients with >70 d. The shortest time was 13 days, the longest was 185 days, the average time was 47.8 days, and the median time was 43.2 days.

### 3.4 Surgery and prognosis

A total of 142 patients underwent stage I intestinal stricture resection and anastomosis. The average postoperative feeding time was 7 days, the longest time was 17 days, and the average postoperative hospital stay was 14 days. All the patients of postoperation have been given total parenteral nutrition (TPN). The TPN was given according to the clinical condition and the duration was 3 days to 21days. Five patients underwent stage I proximal enterostomy, food was administered an average of 4 days after the operation, and patients were discharged from the hospital 10 days after the operation. Three to 6 months after the operation, intestinal stricture resection, and orifice closure were performed. The preoperative rectal mucosa biopsies showed that there were no ganglionic cells in the intestine, and in these cases, patients were treated with radical resection of the megacolon and were discharged from the hospital 10 days after the operation. After the operation, 5 patients had full-thickness infected lacerations of the incision due to malnutrition, which were sutured in the second stage. One patient developed anastomotic fistulas in the pneumoperitoneum, and a colostomy was performed. After discharge, 146 patients were followed-up in the clinic and by telephone, 9 patients demonstrated adhesive intestinal obstructions, 4 patients were treated conservatively, and 5 patients were discharged from the hospital after reoperation. Fifteen patients were admitted to the hospital 1 to 3 times because of recurrent diarrhea that resulted in malnutrition and dehydration. The rest of the children recovered well, their food intake was normal, stools were formed normally, there was no obvious change in watery stool or defecation times compared with normal children of the same age, increases in body mass were not significantly less than those of normal children, and there were no postoperative complications such as adhesive intestinal obstructions.

## 4 Discussion

NEC is a common acute abdominal disease in newborns, especially in premature infants and low birth weight infants. In recent years, with the in-depth understanding and the progression of research for the disease, as well as the development of perinatal neonatal pediatrics, medical knowledge has been greatly improved. Most newborns, including premature infants and low birth weight infants, gradually stabilize after active treatment in the acute phase. However, the incidence of intestinal stenosis in children with NEC has increased annually.^[5,6]^ In contrast to neonatal congenital intestinal stenosis or intestinal atresia, post-NEC strictures are secondary to the disease. Most scholars believe that the repair of intestinal wall ischemic injury caused by inflammatory reactions and intravascular thrombosis is the main pathological mechanism of secondary intestinal strictures or intestinal atresia,^[7]^ especially in children with inflammatory bowel disease.^[8]^ In addition, in intestinal adhesions, the blood vessels that supply the intestine and mesentery are oppressed by adhesive bands, and mechanical pressure leads to interruption of the intestinal wall blood supply, resulting in intestinal wall ischemic necrosis, inflammatory reactions and scar repair. Intestinal adhesions may also be one of the causes of intestinal strictures or atresia.^[9]^ Intestinal strictures caused by scar repair during intestinal injury after NEC are common, and intestinal atresia is rare. In 146 patients who underwent preoperative barium enema, there were 7 cases where barium could not pass through the lesion site, which was indicated to be due to intestinal atresia during the operation, and the rest of the cases were due to intestinal strictures.

Post-NEC strictures are the most common complication in newborns with NEC. Among the 127 children with complete time records, the time of occurrence of intestinal strictures was mainly concentrated during the 4th to 8th week after NEC (62.2%, 79/127), with an average time of 47.8 days and a median time of 43.2 days. This result was much longer than the average 29.7 days reported by Dong et al,^[10]^ which is basically consistent with the results of Schimpl et al.^[11]^ The possible reasons for the difference in time are related to not only the length of fasting time and the tolerance of milk volume^[7]^ but also the NEC stage and the pathological mechanisms of intestinal strictures. The specific reasons need to be further discussed and studied. More than 80% of the intestinal strictures occurred in the colon, and 82.3% (130/158) of the patients had colonic stenosis, which was basically consistent with reports in the literature.^[11]^ In this group, the most common stricture sites were the ascending colon, transverse colon and descending colon, which may be related to the lower blood supply and higher sensitivity to ischemia.^[5]^ It should be noted that many of the intestinal stenoses were multiple stenoses, with an incidence of multiple intestinal strictures of 36.1% (57/158) in this group, and previous reports suggest an incidence as high as 50%.^[11]^ Therefore, during the operation, it is necessary to carefully explore all the segments of the colon, especially in children with primary fistulas, and to carefully explore the distal colon when the mouth is closed. When the adhesion is not clear, saline can be injected into the distal end to determine whether the intestinal passage is clear.

Therefore, all the segments of the colon should be carefully explored during the operation, especially for children with primary colostomy. During colostomy closure, the distal colon should be carefully explored. Sometimes, there are severe adhesions between the intestines, and it is thus difficult to separate the intestines. Saline should then be injected into the distal end to determine whether the intestinal canal is open.

The main clinical manifestations of children with post-NEC strictures were repeated feeding intolerance after oral feeding, such as abdominal distension, vomiting, gastric retention, and haematochezia. The severity of the disease was correlated with the NEC Bell staging. Newborns with stage II and III NEC were more likely to have severe abdominal distension, reduced defecation, and even haematochezia during the course of treatment. Due to the different severities of intestinal strictures and the locations of obstruction, there are substantial differences in the timing of symptoms in infants, resulting in difficulties with diagnosis. The shortest diagnosis time of this group was 12 days, and the longest was 202 days. A previous report found a case of secondary intestinal stenosis that occurred at 11 years old.^[12]^ For infants with ostomy, most of the stricture segments were located at the distal end of the ostomy, and there were no obvious clinical symptoms. In the second stage of the operation, strictures were found in the conventional preoperative examination or during the operation.

Because the main clinical manifestation of intestinal strictures after NEC is digestive tract obstruction, there is no significant difference in clinical manifestations in infants with intestinal obstruction caused by other diseases, but infants with post-NEC strictures have a clear history of NEC.^[13]^ This requires clinicians to ask more questions about past history in suspected infants, especially those referred to the hospital, to avoid misjudgments and delays in treatment.

Abdominal vertical and recumbent lateral X-ray, as a simple and economical method, has a certain reference value for the diagnosis of post-NEC strictures.^[14]^ The imaging findings were fixed dilated intestinal or low intestinal obstructions, and some children showed intestinal rigidity and several instances of gas-liquid flatness. In this group of infants with NEC, X-ray examination was performed when secondary intestinal strictures were suspected, and 88.8% (142/158) of examinations were positive. Gastrointestinal radiography, including upper gastrointestinal radiography and colography, is the first choice for the diagnosis of post-NEC strictures. Because post-NEC strictures mostly occur in the colon, barium perfusion colonography is the first choice.^[15,16]^ In this group, 146 patients were examined by barium perfusion enterography before surgery, 116 patients had suspected intestinal strictures, and the location of stenosis could be judged generally. No obvious abnormalities were found in 18 patients, and total digestive tract angiography was performed in 18 patients. Ileal end obstruction was found in 11 patients, and no obvious positive findings were found in 7 patients. Because of the obvious abdominal distension, laparotomy was performed in combination with the clinical manifestations, and the diagnosis was confirmed to be ileal strictures. In 12 patients with fetal colon, rectal mucosa biopsy was performed for the differential diagnosis. Pathological examination showed that ganglion cells or well-developed ganglion cells could be seen in the submucous membrane of the rectum, and total colon agranulocytosis was excluded. All the children in this group were preterm and low birth weight infants, a finding that was considered to be due to the poor development of the colon, which was not significantly different from that of the narrow segment. Rectal mucosa biopsies were performed in 138 patients, of which 5 biopsies showed a megacolon. The incidence of intestinal strictures caused by megacolon enterocolitis is not high, but this type of stricture should not be ignored. For infants with suspected NEC posterior intestinal strictures and NEC posterior enterostomy, rectal mucosa biopsy is performed routinely before surgery.

Surgery is the only effective treatment for post-NEC strictures. At present, the choice of surgical method is mostly the use of one-stage stricture resection and intestinal anastomosis and, if necessary, intra-intestinal frozen biopsies.^[10,17]^ Intestinal stricture resection and proximal protective enterostomy can also be performed in the first stage, and enterostomal closure can also be performed in the second stage after operation.^[10]^ In addition, with the continuous development of endoscopic technology, laparoscopy has been routinely used in the exploration of post-NEC strictures.^[18]^ The location of the intestinal stricture is very important for the course of operation and the choice of operation method. The location of the intestinal stricture is mainly manifested by the thinning and reduction of the intestinal canal as well as the thickening and stiffness of the intestinal wall. In the narrow part of the intestine, there is obvious fiber exudation and even cable formation outside the serosa as well as adhesions between the intestinal wall and the surrounding area. Of the 158 infants with post-NEC strictures, 142 patients underwent stage I intestinal stricture resection and intestinal anastomosis and recovered well after the operation. The perioperative management of infants with intestinal strictures after NEC is related to the recovery of intestinal function after the operation. As a result of partial colectomy, the loss of water and nutrients easily occurs, resulting in malnutrition as well as water and electrolyte disorders.^[19,20]^ Therefore, adequate parenteral nutrition and enteral nutrition as soon as possible are beneficial for the metabolism, growth and development of patients. After the completion of neonatal surgery, the perioperative treatment plan was made in conjunction with the neonatal internal medicine, nutrition and hepatobiliary surgery departments.

## 5 Conclusion

In summary, intestinal strictures are a common complication of post-NEC strictures, especially in premature infants and low birth weight infants. The possibility of the disease should be taken into account in infants with repeated feeding intolerance, abdominal distension and intestinal obstruction. With the permission of conditions, we should perfect barium enema examination and rectal mucosa biopsy to judge whether stenosis is present and to exclude congenital megacolon. Surgical treatment should be performed timely after a definitive diagnosis because the site of intestinal stenosis is often frequent, and all intestinal segments need to be carefully explored. According to the location and degree of intestinal strictures, most patients should undergo one-stage stricture resection and intestinal anastomosis. If necessary, post-NEC strictures should be resected according to stage, and most post-NEC stricture patients can achieve good therapeutic results and prognoses.

## Acknowledgments

We thank Prof. Chunbao Guo for providing technical assistance during the preparation of the manuscript.

## Author contributions

WL, the first author, contributed to the design of the experiment, the implementation of the experiments, the collection and analysis of the data; ZG, corresponding author, contributed to the design of the experiment, the implementation of the experiment, and the drafting of the manuscript; YW, the second author, contributed to the design of the experiment and the critical reviewing of the specialty knowledge related to the manuscript; JZ, CZ, GL, XW, YS, the third authors, contributed to the collection of the data and provided technical support.
